# Natural Products: Evidence for Neuroprotection to Be Exploited in Glaucoma

**DOI:** 10.3390/nu12103158

**Published:** 2020-10-16

**Authors:** Annagrazia Adornetto, Laura Rombolà, Luigi Antonio Morrone, Carlo Nucci, Maria Tiziana Corasaniti, Giacinto Bagetta, Rossella Russo

**Affiliations:** 1 Preclinical and Translational Pharmacology, Department of Pharmacy, Health and Nutritional Sciences, University of Calabria, 87036 Rende, Italy; annagrazia.adornetto@unical.it (A.A.); laura.rombola@unical.it (L.R.); luigi.morrone@unical.it (L.A.M.); giacinto.bagetta@unical.it (G.B.); 2 Ophthalmology Unit, Department of Experimental Medicine, University of Rome Tor Vergata, 00133 Rome, Italy; nucci@med.uniroma2.it; 3 School of Hospital Pharmacy, University “Magna Graecia” of Catanzaro and Department of Health Sciences, University “Magna Graecia” of Catanzaro, 88100 Catanzaro, Italy; mtcorasa@unicz.it

**Keywords:** glaucoma, neuroprotection, natural products, nutrients, antioxidant, retinal ganglion cells

## Abstract

Glaucoma, a leading cause of irreversible blindness worldwide, is an optic neuropathy characterized by the progressive death of retinal ganglion cells (RGCs). Elevated intraocular pressure (IOP) is recognized as the main risk factor. Despite effective IOP-lowering therapies, the disease progresses in a significant number of patients. Therefore, alternative IOP-independent strategies aiming at halting or delaying RGC degeneration is the current therapeutic challenge for glaucoma management. Here, we review the literature on the neuroprotective activities, and the underlying mechanisms, of natural compounds and dietary supplements in experimental and clinical glaucoma.

## 1. Introduction

Glaucoma is a heterogeneous group of optic neuropathies characterized by typical alterations of the optic nerve head (ONH) and the progressive loss of retinal ganglion cells (RGCs) [1]. The neurodegenerative process often extends along the visual axis affecting the lateral geniculate nucleus (LGN) and visual cortex [2]. In its various subtypes (primary open angle glaucoma, POAG; primary angle closure glaucoma, PACG; normal tension glaucoma, NTG; pseudoexofoliative glaucoma, PEX; etc.), glaucoma is a leading cause of irreversible blindness with more than 112 million people expected to be affected by 2040 [3]. Age and high intraocular pressure (IOP) have been identified as main risk factors; however, NTG occurs in patients with normal IOP values and a portion of patients shows progression even if IOP is pharmacologically maintained in the physiological range [4]. Nevertheless, reducing IOP by hypotonizing drugs or surgical procedures remains the only therapeutic approach currently available [5].

The death of RGCs occurs by apoptosis, and it is the result of several mechanisms including trophic factor deprivation [6], inflammation [7], oxidative stress [8], mitochondrial dysfunction [9], excitotoxicity [10], autophagy dysregulation [11], protein misfolding [12], ischemia, and hypoxia [13]. Each of these pathways may contribute to the etiology and progression of the disease, and it is therefore a potential target for new neuroprotective, IOP-independent, therapeutic approaches. Importantly, these molecular events should not be considered as isolated but rather as a sequence of interconnected mechanisms where sustained oxidative stress acts as key factor [7,8,14]. Indeed, the imbalance between reactive species production (such as reactive oxygen species, ROS, and nitrogen reactive species, RNS), and endogenous antioxidant defenses sustain a vicious cycle that promotes chronic inflammation and creates a hostile environment for neuronal survival.

The retina is particularly susceptible to oxidative stress due to its high consumption of oxygen and proportion of polyunsaturated fatty acids, and its direct exposure to light [15]. This susceptibility increases with aging, due to the physiological decrease of antioxidant defense mechanisms [15]. Furthermore, oxidative stress can also damage the trabecular meshwork (TM) increasing its resistance to aqueous humor outflow and therefore IOP [16].

High levels of oxidative stress markers have been found in aqueous humor of patients with POAG [17] and PACG [18]. Similar results were also reported in plasma of PEX patients [19]. Furthermore, a significant correlation was reported in human TM between oxidative DNA damage, increased IOP, and visual field defects in glaucoma patients [16].

In view of the role played by oxidative stress in predisposing to glaucoma, nutrients and nutraceuticals with antioxidant, anti-inflammatory and anti-apoptotic properties have been extensively studied as a potential, neuroprotective, complementary approach to glaucoma treatment.

Here, we review the current literature on the effect of natural compounds in preclinical and clinical models of glaucoma.

## 2. Vitamins

Vitamins are organic compounds and essential micronutrients found in plants and animals (see Table 1). Vitamins A, D, E, and K are fat-soluble compounds; due to their liposolubility, these vitamins are stored in fatty tissue and liver for days or months [20]. Water-soluble vitamins, such as vitamin C and vitamins B, cannot be stored in the body and need to be replaced more often than fat-soluble ones [21]. The potential neuroprotective effect of vitamins is mainly linked to their antioxidant activity [22,23,24,25].

Several studies assessed the relation between vitamin-rich fruits and vegetables consumption and the risk to develop glaucoma. In the Nurse’s Health Study (NHS) and the Health Professionals Follow-up Study (HPFS) on a cohort of 116,484 participants, no association was reported between dietary antioxidant intake and the risk of POAG [26]. On the contrary, in a cross-sectional study (1155 participants), a higher intake of some vegetables and fruits rich in vitamins A, B2, C, and carotenoids was associated with a decreased risk of glaucoma in old white women [27]. A cross-sectional analysis of the same cohort showed that a higher intake of green leafy vegetables and fruits rich in carotenoids and vitamins A and C was associated with a decreased odd of glaucoma in 662 old African-American women [28]. The prospective Rotterdam Study including 3502 participants aged 55 and older determined no associations between the intake of carotenoids, vitamin C, and vitamin E and the risk of developing glaucoma; vice versa, the group on a high intake of retinol equivalents and vitamin B1 had about a twofold lower risk of open angle glaucoma (OAG) as compared to the group on low intake [29]. A recent cross-sectional study on Japanese American participants, in which a total of 61 out of 581 participants were diagnosed with glaucoma, showed that a high intake of iron and low vitamin A was associated with an increased risk of glaucoma [30].

In a two-year follow up trial, the oral supplementation with vitamins A, B, C, and E, carotenoids, and antioxidant minerals with or without omega-3 polyunsaturated fatty acids (PUFAs) did not prevent visual field deterioration or thinning of the retinal nerve fiber layer in POAG patients [31]. Despite numerous studies, the association between serum vitamin levels and glaucoma prevalence in humans remains controversial. In a cross-sectional analysis (2912 participants) from the prospective National Health and Nutrition Examination Survey (NHANES), Wang and colleagues (2018) evaluated the association between supplement intake and serum levels of vitamins A, C, and E. A significant decrease in the odds of self-reported glaucoma was evident among the group currently using vitamin C supplements, but neither supplement consumption nor serum levels of vitamin A and E were related to glaucoma prevalence [32]. Accordingly, Yuki and collaborators (2010) reported lower serum levels of vitamin C in Japanese glaucoma patients with NTG as compared with controls [33]. NTG risk was not associated with vitamins B6 and B12 or folic acid serum levels [34], and in a meta-analysis study, no correlation was reported between different types of glaucoma and vitamins B6, B12, or D serum levels [34]. On the contrary, in the study by Turgut and colleagues (2010), the plasma levels of vitamin B6 were found to be increased in NTG and POAG patients while no statistical differences were observed for vitamin B12 and folate levels [35].

### 2.1. Vitamin A

Vitamin A, or retinol, is involved in many biological processes such as reproduction, immunity, cell growth, and differentiation, and it is essential for retinal function, since several metabolically active derivatives, including 11-cis-retinal and all-trans-retinoic acid, are required for vision and transcriptional gene regulation, respectively [36]. The results from studies on the association of vitamin A serum levels and glaucoma are several and controversial. Higher concentrations of vitamin A have been reported in patients with POAG as compared to controls [37]. On the contrary, in three other studies, no significant differences were found in vitamin A concentration between glaucomatous and normal patients [33,38,39]. Although the debate is still open, the results of a recent meta-analysis suggested a beneficial association of dietary intake of retinol in open angle glaucoma patients [29].

### 2.2. Vitamin B Complex

Vitamin B1, or thiamin, is an essential cofactor involved in several enzymatic reactions and in many cellular functions [40]. Optic neuropathy is a manifestation, although rare, of thiamin deficiency [41]. The Rotterdam study reported an association between low intake of thiamin and the risk of developing POAG [29]; however, a study on osteoporotic fractures did not find any correlation between vitamin B1 and POAG [27].

Vitamin B2, or riboflavin, is an essential component of two major coenzymes, flavin mononucleotide (FMN) and flavin adenine dinucleotide (FAD). These coenzymes participate in several cellular processes such as electron transport, metabolism of lipids, drugs and xenobiotics, as well as cell growth, development, and signaling [42]. The study by Coleman and colleagues (2008) observed that participants who consumed at least 2 mg/day of vitamin B2 from natural food sources were less likely to be diagnosed with glaucoma [27].

Vitamin B3, or niacin, is the biosynthetic precursor of nicotinamide adenine dinucleotide (NAD^+^) and nicotinamide adenine dinucleotide phosphate (NADP^+^). NAD and NADP are required for the oxidative reaction involved in energy production, and they are substrates for enzymes involved in gene expression, signaling pathways, DNA repair, and cell death [43]. In the nervous system, vitamin B3 plays a key role in neuronal development and survival, and several findings suggest its involvement in neurological deficits and neurodegenerative diseases [43]. Retinal levels of NAD decrease with aging [44], and evidence suggest that therapies increasing NAD levels may be effective against glaucoma [45,46,47]. Williams and collaborators (2017, 2018) demonstrated that diet supplemented with nicotinamide reduced mitochondrial vulnerability and profoundly protected RGCs from degeneration in aged and DBA/2J mice [45,48]. In a recent study, a significantly lower concentration of nicotinamide (the amide form of vitamin B3) was reported in a cohort of POAG individuals as compared to controls [49]. In a crossover, double-masked, randomized clinical trial (57 participants with glaucoma), oral nicotinamide supplementation leads to an early improvement in inner retinal function, which is measured as photopic negative response, in glaucoma patients under IOP-lowering medications [50].

Vitamin B6, or pyridoxine, is a critical cofactor for several biochemical reactions from gluconeogenesis and glycogenolysis to amino acid and lipids biosynthesis and metabolism [51]. Vitamin B6 also plays a role in immune function and neurotransmitter biosynthesis [52]. Furthermore, vitamin B6 can quench ROS [53]. An old Russian study on patients with glaucoma and early stages of cataract reported that eye instillation of low doses of pyridoxine hydrochloride for 20 days affected vision (with changes in visual acuity and enlargement of visual field) and reduced IOP and Becker’s coefficient [54]. Vitamin B6 is responsible for maintaining normal levels of homocysteine, which is an amino acid that generates oxidative stress and induces apoptosis in RGCs [55,56]. Higher levels of homocysteine were detected in the aqueous humor and plasma of POAG patients but not in NTG patients [57,58]. Nevertheless, no clinical correlation has been reported between homocysteine levels and IOP or NTG [59].

Vitamin B9, or folate, is essential for DNA and RNA synthesis; it is also involved in protein metabolism and plays a key role in breaking down homocysteine. Folate is also needed to produce healthy red blood cells and is critical during periods of rapid growth (pregnancy and fetal development) [60]. A reduction of vitamin B9 serum levels was reported in PEX patients [61].

Vitamin B12, also known as cobalamin, is one of the vitamins essential for DNA synthesis and energy production, and it is required for proper red blood cell formation and the integrity of the central and peripheral nervous system [60]. Indeed, deficiency can result in an elevation of homocysteine levels, optic neuropathy, and irreversible damage to the nervous system [62,63]. Tṻrkyilmaz and colleagues (2013) showed that patients with vitamin B12 deficiency have a thinner retinal nerve fiber layer as compared to controls [64]. However, a study by Kang and collaborators (2014) reported no association between vitamin B12 intake and PEX [65].

### 2.3. Vitamin C

Vitamin C, or L-ascorbic acid, is an important physiological antioxidant; besides several other functions, it is an essential nutrient involved in tissue repair and protein metabolism [66]. An old study measured higher levels of vitamin C in the aqueous humor of POAG patients as compared to controls [67], while more recent studies reported opposite results [18,68,69]. Xu and collaborators (2014) described a dose-dependent protective effect of vitamin C against oxidative insult in TM cells that was mediated by the regulation of ROS formation and ion homeostasis, and the activation of autophagy [70]. In vitro studies on TM cells from glaucomatous eyes showed that vitamin C induced the synthesis and reduced the viscosity of hyaluronic acid, increasing humor aqueous outflow through the trabeculum [71,72]. A cross-sectional study on vitamin C supplementation in a population sample in the United States found that consumption of vitamin C was associated with a reduced incidence of glaucoma [39]. Interestingly, a recent study on 1763 volunteers demonstrated that O-methylascorbate, a circulating vitamin C metabolite, has a significant IOP-lowering effect [73].

### 2.4. Vitamin D

Vitamin D, or cholecalciferol, is responsible for the intestinal absorption of several minerals (calcium, iron, magnesium, and zinc) [74]. In addition to calcium homeostasis and bone growth and remodeling, vitamin D is also involved in the regulation of cellular proliferation and differentiation, and immune response [75]. Goncavales and colleagues (2015) reported a positive correlation between vitamin D insufficiency and POAG [76]. A metabolomic analysis on plasma samples from 72 POAG patients showed alterations of vitamin D metabolic pathways as compared to 72 healthy controls [77]; furthermore, the presence of polymorphisms of vitamin D receptor may represent a relevant risk factor for glaucoma development [78]. These data are in agreement with the results of the cross-sectional Korean National Health and Nutrition Examination Survey study (KNHANES), in which a J-shaped association between 25-hydroxyvitamin D (25(OH)D) levels and the risk of POAG was reported [79]. More recently, a case-control study by Ayygari and coauthors (2019) highlighted that patients with advanced glaucoma had lower serum levels of vitamin D as compared with early glaucoma and normal subjects [80]. Accordingly, another two studies showed lower 25(OH)D serum levels in POAG patients [78,81]. At variance, no differences in total vitamin D concentration were found between patients with or without glaucoma in the retrospective cross-sectional Kangbuk Samsung health study [82].

Experimentally, topical administration of 1α,25-dihydroxyvitamin D (3) or its analog 2-methylene-19-nor-(20S)-1α,25-dihydroxyvitamin D (3) significantly reduced IOP in non-human primates [83]. Nevertheless, no variation in IOP values was reported when vitamin D was administered to healthy volunteers with low 25(OH)D levels [84].

### 2.5. Vitamin E

Vitamin E, or alpha-tocopherol, is a potent fat-soluble antioxidant that can prevent the oxidation of polyunsaturated fatty acids (PUFAs) in cell membranes. Furthermore, animal and human studies have shown that vitamin E deficiency impairs immune function [85]. In a rat model of IOP elevation, a correlation between vitamin E deficiency and increased RGCs death with higher levels of retinal lipid peroxidation has been shown [86]. Zanon-Moreno and colleagues (2013) reported lower plasma levels of vitamin E in POAG subjects [87]. Contrariwise, some studies showed an increase in vitamin E serum levels in glaucoma patients [37]. Two studies on NTG patients showed no difference in vitamin E plasma levels between patients with glaucoma and controls [33,39]; vice versa, Lopez-Riquelme and colleagues (2015) reported lower vitamin E plasma levels in NTG patients [38]. In a nonrandomized placebo-controlled study (30 patients), Engin and colleagues (2007) reported the neuroprotective effect of oral supplement of α-tocopherol acetate for 12 months against glaucomatous damage [88].

## 3. PUFAs

Fatty acids (FAs) are carboxylic acids with long aliphatic chains, which constitute the building blocks of cellular lipid structures and play important functional roles as precursors of signaling molecules and energy providers [89]. Based on the number of carbon atoms and unsaturation, FAs can be distinguished in saturated fatty acids (SFAs), monounsaturated fatty acids (MUFAs), and polyunsaturated fatty acids (PUFAs). Alfa-linolenic acid (C18:3n-3) (ALA) and linoleic acid (C18:2n-6) (LA) are the precursor of omega-3 and omega-6 PUFAs, which are essential fatty acids and must be ingested with the diet [90]. LA is the most abundant PUFA in nature, and it can be found in corn, seed oils (i.e., sunflower, soybean, safflower), wheat germ, grape, and hemp; the main dietary sources of ALA are fish, flaxseeds, linseed and canola oils, salmon, tuna and herring [91]. In vivo, ALA is converted in eicosapentaenoic acid (EPA) and docosahexaenoic acid (DHA), and these can be found in seafood, in particular salmon, tuna, sardine, and mackerel and krill oils. DHA is the most important PUFA in the brain [92], and high levels of DHA are found in the retina, mainly in the discs of rods’ external segments [93,94].

In addition to contributing to the physical–chemical properties of cell membranes, with particular reference to the central nervous system and retina [15], PUFAs also serve as precursors of several mediators (eicosanoids, docosanoids) involved in the regulation of inflammation, immunity, blood pressure and viscosity, platelets, cellular growth, synaptic plasticity, etc. [95]. All these actions confer anti-inflammatory, antithrombotic, lipid-lowering, and vasodilating properties to omega-3 PUFAs [96,97,98] that can be exploited for the treatment of human pathologies characterized by a high level of inflammation, including neurodegenerative conditions [99,100,101].

Since a high intake of ALA promotes the production of anti-inflammatory eicosanoids, while a high intake of LA favors the synthesis of pro-inflammatory eicosanoids, the ratio between these nutrients must be balanced [91]. Relating to glaucoma, the association between the intake of omega 3, omega 6, and their ratio, and the incidence of the disease have been evaluated in two studies, the Nurses’ Health Study and Health Professionals Follow-up Study and the prospective SUN (Seguimento University of Navarra) cohort study; the results showed that participants with a high omega 3:6 ratio intake had a higher risk of developing glaucoma [102,103]. Conversely, in a cross-sectional population study (3865 participants), enhanced daily intake of EPA and DHA were linked with significantly lower risks of glaucoma. However, daily levels of total PUFAs intake in the higher quartiles were associated with a higher risk of glaucoma; this was probably due to the relative intake of omega-3 and omega-6 and the presence of other confounding comorbidities [32].

In view of their mechanisms of action, preclinical and clinical studies on the effect of PUFAs intake in glaucoma degeneration have either focused on its effects on IOP levels or RGC function preservation.

An increased consumption of omega-3 has been shown to decrease IOP in a rat model due to the production of prostaglandins (PGs) that possess IOP-lowering effects [104]. Interestingly, a recent clinical study showed that oral omega-3 supplementation for three months in normotensive adults significantly reduced IOP [105].

It has been suggested that a dietary combination of omega-3 and omega-6 PUFAs given for 6 months is more effective than single supplementation; in fact, a diet supplementation with a combination of EPA, DHA, and gamma linolenic acid given for 6 months prevented retinal cell structure damage and decreased glial cell activation induced by the laser-induced chronic elevation of IOP in rats [106]. A dietary deficiency in omega-3 affects retinal function with an alteration of RGCs activity, while diets rich in omega-3 help reduce the dysfunction of RGCs induced by acute IOP elevation in rats [107,108]. Inman and collaborators (2013) reported that the dietary administration of ALA to glaucomatous DBA/2J mice led to a significant reduction in RGCs loss and dysfunction [109]. Recently, it was also reported that omega-3 PUFAs supplementation potentiated the neuroprotective effect of timolol treatment in retinas of DBA/2J mice [110].

Despite the preclinical positive results on PUFA supplementation, an open-label randomized controlled trial on 117 patients with mild or moderate POAG and IOP under control with topical medications failed in reporting any benefit after oral antioxidant supplementation (mixture of vitamins and minerals) with or without omega-3 PUFAs [31].

In view of the examined literature, the role of PUFAs as a potential treatment in glaucoma remains controversial and justifies further investigation and longer-term research with a larger sample and/or with different PUFAs combinations.

## 4. Palmitoylethanolamide

Palmitoylethanolamide (PEA) is a lipid mediator synthesized during inflammation and tissue damage endowed with neuroprotective, anti-neuroinflammatory, and analgesic properties [111]. It was isolated from tissues, body fluids, and purified lipid fractions of egg yolk, soybeans, and peanut meal, and it has been found in a wide variety of food [111]. At a dosage of 600/1200 mg/d, PEA is marketed as a medical food in several European countries, and it is used as a dietary supplement in ocular diseases in Italy [112]. This lipid mediator is synthetized by the hydrolysis of the phospholipid precursor, N-palmitoyl-phosphatidyl-ethanolamine, by N-acyl-phosphatidyl-ethanolamine-selective phospholipase D (NAPE-PLD) [113], and it is degraded to palmitic acid and ethanolamine by two different hydrolytic enzymes, the fatty acid amide hydrolase (FAAH) [114] and the N-acylethanolamine-hydrolyzing acid amidase (NAAA) [115].

Its use as a dietary supplement in glaucoma was validated by different clinical data, while preclinical studies have investigated its mechanism of action highlighting an entourage effect with the endocannabinoid system [111]. This system includes the endogenous ligands N-arachidonoylethanolamide (anandamide, AEA) and 2-arachidonoylglycerol (2-AG), which are the enzymes responsible for their synthesis and degradation, such as fatty acid amide hydrolase (FAAH) and monoacylglycerol lipase (MGL), and the cannabinoid type-1 (CB1) and type-2 (CB2) receptors [116]. AEA and 2-AG also bind to GPR55 [117], transient receptor potential vanilloid 1 (TRPV1) ion channel [111], and nuclear peroxisome proliferator-activated receptors (PPARs) α and γ [118]. Early evidence indicate that the levels of PEA and 2-AG decreased significantly in eye tissues of glaucomatous patients as compared to normal donors [119], suggesting that the lipid mediators have a role in this ocular disease.

Patients with ocular disease showed protective effects after an intake of PEA as documented in clinical trials. Oral intake of PEA (600 mg/day) for fifteen days prevented the significant increase of postoperative IOP in patients who had undergone bilateral laser iridotomy as compared to those pretreated with placebo [120]. After three months of PEA oral intake, a reduction of IOP and a significantly improvement endothelial function were observed in ocular hypertension (OHT) patients as compared to placebo-treated. Interestingly, this effect lasted longer than the period of PEA administration [121]. A significant reduction of IOP values was observed in POAG and OH patients after oral administration of PEA for two months [122]. In NTG patients, Costagliola and colleagues (2014) demonstrated that the systemic administration of PEA for six months reduced IOP and improved visual field indices; no ocular or systemic side effects were recorded after this longer lasting treatment [123]. Recently, Rossi and colleagues (2020) demonstrated that the oral administration of PEA (600 mg/day) for four months enhanced the electric activity of RGCs and retina, which was measured by pattern evoked electroretinograms (PERG), and improved IOP [124]. These clinical data, together with preclinical results, have led to speculate that PEA has at least three potential beneficial effects in patients with glaucoma [124]: (1) increase of aqueous humor outflow through the GPR55 and the PPARα receptors and the involvement of the p42/44 mitogen-activated protein kinase (MAPK) pathway [112]; (2) vasorelaxation of the ophthalmic artery by acting on the transcription factors PPARα [125]; and (3) engagement of the cannabinoid system, which has been shown to mediate neuroprotective effects in both the central nervous system [126] and eye [127]. In a rat model of ischemia reperfusion injury, AEA reduced glutamate excitotoxicity and prevented apoptosis by activating CB1 and TRPV1 receptors [128,129,130]. An interesting hypothesis is that PEA could compete with AEA for the FAAH active site, increasing AEA concentration and its neuroprotective effects [111].

## 5. Melatonin

Melatonin (N-acetyl-5-methoxytryptamine) is an indolamine secreted mainly by the pineal gland and known as a regulator of the circadian rhythm in mammals. Melatonin synthesis also occurs in several ocular structures, such as lachrymal glands, lens, ciliary body, and retina. The hormone exerts paracrine and autocrine effects that are either mediated by its G-protein coupled receptors, MT1 and MT2, and the putative MT3, or are receptor-independent [131]. In the eye, melatonin locally synthesized or entering from the circulation participates in the regulation of retinomotor movements, rod outer segment disc shedding, dopamine synthesis, and intraocular pressure homeostasis [132]. Moreover, due to its antioxidant and free radical scavenger properties, melatonin protects ocular tissue from oxidative stress induced by light. Indeed, melatonin is an effective antioxidant and a potent free radical scavenger acting on either ROS or RNS [133]. Moreover, its antioxidant activity is potentiated by its ability to (1) stimulate endogenous antioxidant (such as glutathione peroxidase and superoxide dismutase); (2) downregulate prooxidant enzymes; and (3) increase mitochondrial oxidative phosphorylation efficiency and reduce electron leakage (therefore reducing free radical generation). In view of its properties and also considering its lipophilic nature, which enables melatonin to easily cross the hematoencephalic and hematoretinal barriers, its ability to mitigate cell damage has been explored for several neurodegenerative diseases, including glaucoma [134].

In a rat model of chronic hypertension induced by the injection of hyaluronic acid in the anterior chamber of the eye, a subcutaneous implant of a melatonin-containing pellet reverted the alteration of retinal function and reduced RGC vulnerability; ex vivo experiments in explanted retinas demonstrated that exposure to melatonin modulated glutamatergic, GABAergic (gamma aminobutyric acid), nitrergic transmissions, and retinal redox status, suggesting that reduced excitotoxicity and antioxidant effects were involved in the neuroprotection observed in vivo [135]. Accordingly, an intravitreal injection of melatonin exerted neuroprotective and antiapoptotic effects in a rabbit model of retinal oxidative toxicity induced by glutamate [136].

In a mouse model of transient retinal ischemia, Park et al. showed that melatonin (intraperitoneally injected one hour before, at the time of ischemia, and one hour after) inhibited the increased expression of hypoxia-inducible factor-1α (HIF-1α), reduced Muller cells activation, and prevented RGC death [137]. The protective effects of melatonin against hypoxic damage in neonatal retina were reported by Kaur and colleagues (2013); the study showed that melatonin treatment prevented RGC apoptosis, reduced lipid peroxidation, increased retinal glutathione (GSH) content, suppressed microglial expression of pro-inflammatory cytokines (i.e., TNF-α and IL-1β), and decreased vascular permeability [138].

Interestingly, in a model of optic nerve (ON) transection, pinealectomy exacerbated the retrograde degeneration of RGCs while an intraperitoneal injection of melatonin protected axotomized RGCs; the exogenous administration of melatonin was effective only in pinealectomized, melatonin-deficient mice but not in non-pinealectomized animals [139], thus suggesting that the supplementation may be suitable for protection under conditions of melatonin deficiency.

In addition to its neuroprotective properties, melatonin also shows hypotensive direct effects on IOP. Oral or topical administration of melatonin reduced IOP in rabbits [140,141,142] and monkeys [143]. The topical administration of melatonin decreased IOP in normotensive control and glaucomatous DBA/2J mice via the MT2 receptor [144].

In human normotensive subjects, Samples et al. found that oral melatonin reduced IOP by about 10% [145]. IOP reduction was reported in patients that underwent cataract surgery when treated with melatonin [146]. More recently, a short-term prospective study showed a reduction of IOP value in normotensive subjects taking a melatonin-based supplement [147]. However, the study lacked a placebo group, and the reported IOP reduction was only limited to 1 mmHg. Pescosolido and colleagues (2015), in a pilot study on 10 POAG patients treated with multiple hypotensive topical drugs, reported that oral treatment with agomelatine, a melatonin analogue, was able to significantly further reduce IOP [148].

Interestingly, mice lacking the MT1 receptor have elevated IOP at night and a reduced number of RGCs [149,150], and melatonin blood levels are altered in patients with glaucoma [151], suggesting that dysfunctional melatonin signaling may represent a possible risk factor for glaucoma.

## 6. Citicoline

Citicoline (cytidine-5′-diphosphocholine, CDP-choline) is a naturally occurring endogenous compound acting as a choline donor. Indeed, after oral administration, citicoline bioavailability is higher than 90%, and it is fast metabolized to cytidine and choline [152]. Citicoline functions as an intermediate in the biosynthesis of cell membrane phospholipids and as a precursor of the neurotransmitter acetylcholine [153]. As a dietary supplement, citicoline has been used as a neuroprotective agent in several neurological disorders, including Parkinson’s and Alzheimer’s diseases, dementia, stroke, and glaucoma [154,155,156,157]. The neuroprotective effects of citicoline are mediated by several mechanisms such as the maintenance of sphingomyelin and cardiolipin levels (a component of the inner mitochondrial membrane essential for mitochondrial electron transport), restoration of phosphatidylcholine levels, increased activity of glutathione synthesis, reduction of lipid peroxidation, and attenuation of free fatty acid release [158]. Furthermore, citicoline increases acetylcholine, dopamine, noradrenaline, and serotonin levels in several brain regions [159,160] and dopamine release in retina [161].

The neuroprotective properties of citicoline have been shown in several paradigms of experimental glaucoma. In a mouse retinal explant, citicoline exerted antiapoptotic effects on damaged RGCs (by reducing caspase-9 and caspase-3 activity) and supported axon regeneration [162]. After partial ON crush, intraperitoneal treatment with citicoline protected RGCs and their axons from delayed neurodegeneration and increased retinal expression of the antiapoptotic protein Bcl-2 [163]. In rat primary retinal cultures exposed to glutamate or high glucose, citicoline exerted antiapoptotic effects and reduced synaptic loss [164]. Following an intravitreal injection of kainic acid in rat, intraperitoneal injection of citicoline prevented changes of retinal thickness and attenuated the upregulation of nitric oxide synthase (NOS) isoforms [165]. In the same animal model, Park and colleagues (2006, 2007) reported a reduction of extracellular signal-regulated kinase 1/2 (ERK1/2) activation and clusterin expression [166,167].

The clinical efficacy of citicoline treatment in glaucoma patients has been proven by several trials. Intramuscular or oral administration of citicoline for two 60-day periods has been associated with an improvement of retinal function (evaluated by pattern electroretinogram recording, PERG) and neural conduction along the visual pathway (evaluated by visual evoked potential recordings, VEP) in glaucoma patients with moderate visual field defects [168]. An extension of citicoline treatment up to 8 years prevented the regression observed after 120 days from treatment suspension and stabilized the improvement of VEP and PERG parameters [169]. The study by Ottobelli and colleagues (2015) confirmed the long-term beneficial effect of oral supplementation with citicoline, suggesting that the treatment might significantly slow down the progression rate of glaucoma [170]. Furthermore, topical treatment with citicoline eyedrops in open angle glaucoma patients was effective in enhancing PERG amplitude and improving VEP parameters [171]. Last, the results of a pilot randomized placebo-controlled clinical trial on 80 patients with mild to moderate OAG (primary or PEX) receiving citicoline eyedrops or placebo for 3 years suggest that citicoline might reduce disease progression in patients with IOP < 18 mmHg [172].

## 7. Coenzyme Q10

Coenzyme Q10 (CoQ10), or ubiquinone, is an important cofactor of the mitochondrial electron transport chain and a potent lipid-soluble antioxidant [173]. CoQ10 acts by maintaining the mitochondrial membrane potential, sustaining ATP synthesis, and preventing ROS generation [174,175]. Several in vitro and in vivo studies support the neuroprotective effect of CoQ10 in retinal diseases [176]. In a rat model of transient IOP elevation, the topical administration of CoQ10 decreased extracellular glutamate levels, minimized retinal damage, and prevented apoptotic cell death [177,178]. The antiapoptotic effects of CoQ10 may involve the inhibition of the mitochondrial permeability transition pore (MPTP) opening that prompts the apoptosis intrinsic execution pathway [179]. Ju and colleagues (2018) tested the effect of a diet supplement with ubiquinol (the reduced form of CoQ10) in a mouse model of retinal ischemia/reperfusion injury; the treatment prevented apoptotic cell death and enhanced RGC survival by modulating the Bax/Bad/Bcl-xL pathway [180]. Diet supplementation with CoQ10 for 6 months significantly promoted RGC survival and preserved RGC axons in the lamina cribosa in glaucomatous DBA/2J mice; CoQ10 treatment, as compared to a control diet, reduced the upregulation of NMDA receptor subunit type 1 (NR1), NMDA receptor subunit type 2A (NR2A), SOD2, and heme oxygenase-1 (HO-1) protein expression and prevented alteration of the mtDNA content [181]. Interestingly, an in vitro study testing the effects of H_2_O_2_-induced oxidative stress in ONH astrocytes demonstrated that CoQ10 treatment triggered mitochondrial biogenesis and preserved mitochondrial morphology and mass while maintaining oxidative phosphorylation system (OXPHOS) protein expression and cellular ATP production [182].

Due to the large molecular weight and hydrophobicity, topical CoQ10 has a poor intraocular penetration and bioavailability [183]. Furthermore, this compound is a substrate of the P-glycoprotein (P-gp), which is an efflux membrane transporter expressed on corneal epithelial cells and RGCs [184]. For these reasons, CoQ10 is usually used in combination with vitamin E, which ameliorates the bioavailability of CoQ10 by inhibiting P-gp [185]. Davis and collaborators (2017) observed that the formulation of CoQ10 into micelles using the vitamin E derivative D-α-tocopherol polyethylene glycol 1000 succinate (TPSG) were neuroprotective in primary mixed retinal cultures exposed to mitochondrial damage [184]. An in vivo topical application of CoQ10 micelles prevented RGC apoptosis in a rat model of ocular hypertension (OHT) and following mechanic ON [184,186].

In a randomized clinical study on 64 PEX patients treated with a topical combination of vitamin E and CoQ10 (CoQun^®^), a reduction of oxidative stress, measured by decreased levels of SOD in the aqueous humor, was reported [187]. In a pilot study enrolling 43 OAG patients in monotherapy with β-blockers, Parisi and collaborators (2014) reported a beneficial effect on the inner retinal function, with consequent enhancement of the visual cortical responses, in patients treated with CoQun^®^ drops (instilled twice daily) for 6 or 12 months [188]. Currently, a multicenter controlled clinical trial on 612 POAG patients has been designed to evaluate the efficacy of topical combination of vitamin E and CoQ10 (CoQun^®^) [189].

## 8. Taurine

Taurine (2-aminoethylsuphonic acid), a sulfur amino acid synthesized in the liver of most mammals, is present in high levels in tissues such as muscles and eyes (mainly in the retina), and it is mostly obtained from diet (e.g., meats, seafood and fish) [190]. Intake of the amino acid from dietary sources is highly dependent on taurine transporter expression [191]. In retinal cells, taurine uptake was demonstrated in retinal pigment epithelium, retinal glial cells, RGCs, and cell photoreceptors [192]. Nutritional taurine depletion has been shown to cause photoreceptor degeneration in cats [193], monkeys [194] and dogs [195], suggesting that taurine endogenous synthesis can be insufficient. Using a metabolomic approach, low levels of taurine were measured in patients with POAG as compared with age- and sex-matched non-POAG controls. The crucial role of taurine in retinal protection was evident in epileptic patients treated with vigabatrin, showing that retinal phototoxicity correlated with insufficient taurine plasma levels [196]. Retinal degenerative effects were observed in rats treated with taurine transport inhibitors, such as β-alanine or guanidoethane sulfonate (GES) [197,198].

It has been suggested that taurine supplementation may have a neuroprotective effect in retina [199]. Accordingly, taurine improved RGC survival in a rat retinal explant exposed to serum deprivation or N-methyl-D-aspartate (NMDA) [200]. The same authors observed the protective effects of taurine intake in DBA/2J mice and rats with episcleral vein occlusion and in a model of retinitis pigmentosa with secondary RGC degeneration [200]. Furthermore, the antiapoptotic effect of taurine was recently reported against NMDA-induced retinal excitotoxicity in rats [201,202,203].

Although the mechanism underlying the protective effect of taurine on RGCs is not completely clarified, it was suggested that it inhibits NADPH oxidases, which are the primary source of superoxide induced by NMDA receptor activation [204]. Successively, it was demonstrated that the selective blockade of taurine transporter prevented the taurine-mediated increase of RGC survival, suggesting that the neuroprotective effects of taurine are mediated by its intracellular/mitochondrial action [205].

Taurine is structurally related to the neurotransmitter gamma aminobutyric acid (GABA); therefore, it has been suggested that its neuroprotective effects could be mediated by the interaction with GABA receptors [190]. Indeed, GABA-B receptors antagonist prevented the taurine-elicited neuroprotective effect in RGCs [205].

## 9. Flavonoids

Flavonoids are secondary metabolites of plants, which are generally categorized as phenols or polyphenols. The classification of flavonoids is based on the level of oxidation in the ring structure; however, major dietary flavonoids are often classified under six groups (i.e., anthocyanidins, flavonols, flavanols, isoflavonoids, flavones, and flavonones) [206].

Over 4000 flavonoids have been identified. High concentrations of flavonoids are present in fruits and vegetables but also in red wine and chocolate [207]. These compounds have been shown to exert antioxidant and anti-inflammatory effects in different pathological states, including cancer, cardiovascular diseases, and neurodegenerative disorders [208]. Clinical data analyzed in systematic reviews suggest that flavonoid intake can help in maintaining or restoring the visual field in patients with OHT or glaucoma, although no significant effects were observed on IOP values [209,210]. In particular, the main effects were reported with anthocyanins, Ginkgo biloba, and green tea.

### 9.1. Ginkgo Biloba Extract (GBE)

*Ginkgo Biloba* extract (GBE) is obtained from the leaves of *Ginkgo Biloba*, which is an ancient Chinese tree. The extract contains several chemical constituents, including “flavones glycosides”, such as quercetin, kaempherol, and isorhamnetin, and “terpene lactones”, such as ginkgolides A, B, C, and bilobalide. Many types of GBEs are found on the market, but clinical and preclinical studies have been performed mainly using the standardized EGb 761 containing flavonoids (24%), terpene lactones (6%), and a low concentration of ginkgolic acids (0.0005%), which is known to cause allergenic and genotoxic effects. EGb761 is used for ailments associated with aging, such as neurodegenerative disorders, cognitive decline, vascular insufficiency [211], and glaucoma [212].

Neuroprotective effects of Ginkgo biloba were observed in different animal models of glaucoma [212]. In the episcleral vein cauterization model, treatment with EGb 761 for 5 months induced a significant reduction of RGC loss [213]. Similar results were observed in a model of ON crush following four weeks of treatment with the extract [214,215,216].

Clinical studies on GBE were based on two different outcomes, blood flow or visual field improvement [212]. Park et al. (2011) observed an increase of peripapillary blood flow in patients with NTG after 4 weeks of GBE (80 mg twice daily) oral administration [217]. The effects of GBE on ocular blood flow were confirmed in patients with OAG treated for four weeks with a rich antioxidant dietary supplement containing 120 mg/day of GBE [218]. Supplementation with GBE (40 mg three times daily) for 4 weeks induced a statistically significant improvement in visual field indices and pre-existing progressive visual field deficits in Italian patients with NTG when compared with placebo; no differences in IOP or blood pressure were reported [219]. Positive results were observed also by Shim and colleagues (2012) that measured an improvement of the visual function in individuals with NTG after oral intake of Bilberry anthocyanins and GBE [220]. However, a different study in Chinese patients with NTG treated with the same posological scheme of GBE reported no significant improvements in visual field defects [221]. A possible explanation for these contrasting results may reside in the variation of study population and racial differences [212].

### 9.2. Green Tea

Tea beverage, made from the infusion of the leaves of *Camellia sinensis,* contains polyphenols, i.e., flavanols (also known as catechins), alkaloids (such as caffeine and theobromine), carbohydrates, tannins, and minerals (such as fluoride and aluminum). The catechins present in green tea are (-)-epigallocatechin-3-gallate (EGCG), (-)-epigallocatechin (EGC), (-)-epicatechin-3-gallate (ECG), and (-)-epicatechin (EC). Other food rich in catechins are red wine, dark chocolate, legumes, and nuts. EGCG is the major catechin and has been used for most of the research carried out to investigate the health effects of catechins [222]. A clinical study showed that in OAG patients with early to moderate ON damage, oral treatment with EGCG (200 mg/day) induced a small but significant effect on inner retinal function evaluated by pattern evoked electroretinograms (PERG) [223]. A significant increase in the a- and b-wave amplitudes of the electroretinogram (ERG), other than a decrease of the apoptotic pathway, was observed in an in vivo preclinical study using a retinal ischemia/reperfusion model [224]. Neuroprotective effects on RGCs were shown in the ON crush model after pre and post systemic treatment with EGCG [225]. The neuroprotective effects of catechin (50 mg/kg, intraperitoneal (i.p.)) reported by Peng and colleagues (2008, 2010) in ischemia/reperfusion (I/R) and optic nerve axotomy models were associated with a decrease of nitric oxide expression and upregulation of the Akt/PI3kinase pro-survival pathway [226,227].

The protective effects of green tea extract (GTE) in toto was recently investigated. Pharmacokinetic studies, performed in rats, indicated that after oral intake of GTE, catechins (e.g., EGCG, EGC, and EC) are quickly distributed into various eye compartments and exert pro- and antioxidative effects depending on the tissues and EGCG concentration [228]. Yang et al. (2019) demonstrated that an intragastric administration of GTE, 4 times/day with a dose of 275 mg/kg, ameliorated ischemia-induced RGC degeneration and prevented RGC function impairment through antiapoptotic, antioxidant, and anti-inflammatory effects [229].

## 10. Resveratrol

Resveratrol (3,5,4′-trihydroxystilbene) is a polyphenol, first isolated from the roots of white hellebore (*Veratrum grandiflorum O. Loes*) and *Polygonum cuspidatum.* It is found in vegetables and fruits, including berries, grapes, and peanuts, and in red wine. It has been shown that resveratrol can slow down the progression of a wide variety of illnesses, including cancer, cardiovascular, neurodegenerative disease, and ocular diseases [230].

Several preclinical studies investigated the health benefits of this polyphenol in different glaucoma models. Oral resveratrol administration (250 mg/kg/day for four weeks) reduced RGC loss and preserved pupillary light response following ON crush injury [231]. The same authors by using mice with conditional deletion of sirtuin-1 (SIRT-1) in neurons demonstrated the involvement of SIRT-1 activation in the resveratrol-mediated RGC neuroprotection [231]. Dietary supplementation for 11 months with a low dose of resveratrol (31 mg/kg/day) afforded neuroprotection in RGC injury induced by ON nerve crush, increasing the expression of the cytoplasmic binding immunoglobulin protein (BiP), nuclear C/EBP homologous protein (CHOP), and nuclear X-box binding protein-1 (XBP-1) [232]. In a model of chronic OHT, Zhang et al. (2018) observed that the intragastric administration of resveratrol (20 mg/kg/d) for 4 weeks prevented retinal damage and RGC apoptosis by activation of the AMP activated protein kinase/peroxisome proliferator-activated receptor-c coactivator-1α (AMPK/PGC-1α) signaling pathway and upregulation of mitochondrial proteins [233].

In the ischemia/reperfusion model, intraperitoneal (i.p.) administration of resveratrol reduced the decrease of retinal thickness and preserved cellular density in ganglion cell layer (GCL) via the downregulation of caspase-3 and caspase-8 expression and suppression of gliosis-related inflammation [234,235]. Recently, Cao and colleagues (2020) observed that the intravitreal administration of resveratrol (300 µM) delayed RGC apoptosis in a model of transient IOP elevation. The reported neuroprotection was associated with a decreased generation of ROS and expression of acetyl-p53, and an upregulation of brain-derived neurotrophic factor (BDNF) and its tyrosine receptor kinase B (TrkB) receptor [236]. These preclinical data suggest that resveratrol induces protective effects by modulating oxidative stress as well as apoptotic and inflammatory pathways. This hypothesis is supported by in vitro experiments in retinal cell lines. Photoreceptor death induced by glucose deprivation (GD) was inhibited by the polyphenol through the inhibition of caspase-9 and caspase-3 and elevation of SIRT-1 expression and activity [237]. In glaucomatous human TM, treatment with resveratrol prevented the increase of IL-1α, IL-6, IL-8, ROS, and endothelial-leukocyte adhesion molecule 1 (ELAM-1) and reduced the expression of the senescence markers sa-β-gal, lipofuscin, and the accumulation of carbonylated proteins [238,239]. Anti-inflammatory effects of resveratrol were confirmed in cultured ONH astrocytes where the treatment reduced iNOS [239] and decreased PGE_2_ receptor expression [240].

## 11. Forskolin

Forskolin is a diterpene (7beta-acetoxy- 8,13-epoxy-1alpha,6beta,9alpha-trihydroxy-labd-14-en-11-one) extracted from the roots of Coleus Forskohlii (Lamiaceae). The cellular effects of forskolin rely on its ability to activate the catalytic subunit of adenylate cyclase, increasing the intracellular levels of cAMP [241].

Forskolin has been shown to be effective in lowering IOP in rabbits, monkeys [242,243,244], and humans [242,243,244,245,246]. In an open label pilot study enrolling 16 POAG patients, supplementation with forskolin, rutin, and vitamins B1 and B2 potentiated the hypotonizing effects of pharmacological treatments [247]. Similarly, in a randomized controlled trial (97 subjects), oral administration of forskolin and rutin was associated with a better control and a further reduction of IOP in POAG patients poorly responsive to multitherapy treatment [248].

More recently, Mutolo and colleagues (2016) reported a reduction of IOP values and an improvement of PERG amplitude in POAG patients under IOP-lowering medications and treated with a combination of forskolin, homotaurine, carnosine, and folic acid [249].

In vitro and in vivo evidence suggested that forskolin protects RGCs against glaucoma-related insult. Some of these neuroprotective effects seem to be mediated by the activation and potentiation of neurotrophins’ activity [250,251]. Indeed, the addition of forskolin to brain-derived neurotrophic factor (BDNF), ciliary derived neurotrophic factor (CTNF), and insulin-like growth factor-1 (IGF-1) in culture medium promoted RGC survival [251,252]. Similarly, when added to a combined treatment with BDNF and CTNF, forskolin significantly improved the survival of axotomized RGCs in cat retina [250,253]. Forskolin partially prevented RGC death in a rat model of retinal ischemia/reperfusion injury, and this effect was potentiated by the simultaneous administration of homotaurine and L-carnosine [254]. The observed neuroprotection was associated with reduced calpain activity and upregulation of the phosphoinositide-3-kinase/protein kinase B (PI3K/Akt) pathway, while it was insensitive to protein kinase A (PKA) inhibition and independent from the hypotensive action of forskolin [254]. More recently, the protective efficacy of a dietary supplement containing forskolin, homotaurine, spearmint, and vitamins B was reported in a mouse model of ON injury, and following a transient increase of IOP, the supplement intake reduced RGC loss, preserved ERG photopic negative response, and reduced pro-apoptotic markers and inflammatory cytokines expression [255,256].

## 12. Curcumin

Curcumin is a polyphenolic orange and water-insoluble pigment isolated from turmeric, the rhizome of *Curcuma longa* L., belonging to the Zingiberaceae family. This spice has been widely used as food flavoring and coloring agent and, in Asian countries, as herbal medicine [257].

Curcumin has been reported to attenuate cognitive deficits, neuroinflammation, and plaque pathology in Alzheimer’s disease models [258], and it showed neuroprotection in cerebral ischemia [259] and in neuronal cultures exposed to excitotoxic stimuli [260].

Several pharmacological properties have been attributed to curcumin including antimicrobial, anti-inflammatory, antioxidant, antimutagenic, and anticancer activities [261,262]. Indeed, it has been shown that curcumin effectively inhibits lipid peroxidation and ROS, decreases inflammatory cytokines, reduces oxidative stress, and increases antioxidant enzymes in age-related eye disease [263,264].

The mechanisms by which curcumin induces its effects are yet to be fully elucidated; however, it can modulate the expression and activation of proteins involved in the inflammatory response (i.e., chemokines, interleukins, and transcription factors). Curcumin attenuates mitochondrial-mediated oxidative stress [265], downregulates cyclooxygenase-2 (COX2) and inducible nitric oxide synthase (iNOS), reduces astrogliosis by downregulating the janus kinase 2 (JAK2)– signal transducer and activator of transcription 3 (STAT3) pathway [266] and possesses antiangiogenic activity via modulation of the vascular endothelial growth factor (VEGF)/VEGF receptor (VEGFR)/K-Ras pathway [267]. Several studies identified nuclear factor- kB (NF-kB) as one of the mediators for curcumin’s effects [268,269], while others suggest that curcumin may exert anti-inflammatory effects through the activation of peroxisome proliferator-activated receptor-gamma (PPAR-γ) [270]. A recent meta-analysis of randomized controlled trials showed that curcumin improves systemic markers of oxidative stress and increases the serum activities of antioxidant enzymes such as SOD, catalase, and glutathione peroxidase [271].

It has been shown that the antioxidant and anti-inflammatory properties of curcumin may protect RGCs from glaucoma-related stimuli. Curcumin prevented NMDA-induced apoptosis in mixed rat retinal cultures [272]. Interestingly, curcumin-mediated neuroprotection against excitotoxicity was correlated with an increased level of the NMDA receptor subunit type 2A (NR2A) and decreased amplitude of NMDA currents [273].

Supplementation of rodent diet with 0.01% to 0.25% curcumin protected RGCs and microvessels from the damage induced by ischemia/reperfusion insult through its inhibitory effects on NF-kB and STAT3 activation and monocyte chemoattractant protein-1 (MCP-1) upregulation [274].

In a rodent model of OHT induced by episcleral veins cauterization, intragastric administration of curcumin (10 mg/kg/day) for 6 weeks significantly inhibited the death of RGCs [275]. However, the dosage reported in these experimental models is equal to 800 mg/day in humans, which has been associated to side effects such as nausea, diarrhea, and the upregulation of lactate dehydrogenase and alkaline phosphates serum levels [276]. The systemic therapeutic treatment with curcumin is further challenged by its limited oral absorption and systemic availability [277]; furthermore, the extremely low water solubility would represent an obstacle for topical administration in ophthalmic diseases. To overcome these limitations, Davis and colleagues [261] developed a novel nanocarrier formulation of curcumin in D-α-tocopheryl polyethylene glycol succinate (TPGS)/Pluronic F127 that significantly increased curcumin solubility (4.3 mg/mL). This formulation was found to be neuroprotective in R28 retinal cultures exposed to glutamate or cobalt chloride (hypoxia mimetic), and given topically twice daily for three weeks (starting two days prior induction of the insult), it significantly preserved RGC density in a rat model of OHT and following partial ON transection [261]. Pursuing the aim to overcome the pharmacokinetic limitations of curcuma, increase its penetration into aqueous humor, and guarantee a sustained and prolonged release, while reducing systemic toxicity, a more recent study developed a thermosensitive hydrogel containing latanoprost and curcumin-loaded nanoparticles for topical eyedrop formulation (application) [278]. Treatment with the hydrogel of human TM cells exposed to H_2_O_2_ prevented the cellular damage induced by oxidative stress via decreasing inflammation-related gene expression (TNF-α, IL-1α), mitochondrial ROS production, and caspase-3 activity [278]. Accordingly, a previous study showed that the antiapoptotic effect of curcumin in TM cells exposed to oxidative stress was mediated by the inhibition of pro-inflammatory factors (i.e., IL-6, ELAM-1, IL-1alfa, and IL-8), reduction of carbonylated protein levels, and decrease of the senescence marker SA-β-gal activity [279].

## 13. Lycium barbarum

*Lycium barbarum L.* is a Solanaceous defoliated shrub widely distributed in northwestern China; the plant also grows in southwestern Europe and Mediterranean areas. The *Lycium barbarum*’s fruit, called wolfberry or goji berry, has long been known in China and other Asian countries as traditional herbal medicinal and food supplement; more recently, it has becoming very popular in Western countries. Health claims for *Lycium barbarum* are anti-aging effects, balance of the immune system, improvement of lung, kidney, and liver function, and preservation of vision [280,281,282]. The fruits of *Lycium barbarum* contain several bioactive compounds including abundant polysaccharides (LBP), polyphenols/flavonoids, carotenoids (zeaxanthin and β-carotene), amino acids, vitamins (in particular riboflavin, thiamin and ascorbic acid), fatty acids, and trace of zinc, iron, and copper. The most active component of the fruit has been identified in the polysaccharide components [283]. LBP (i.e., water-soluble conjugates including rhamnose, xylose, glucose, mannose, arabinoside, and galactose) accounts for 5–8% of dried fruit and about 40–45% of water fraction; it is endowed with a wide array of pharmacological activities (or is responsible for most of the health benefits mediated by the berry) including anti-aging, cytoprotective, antioxidant, anti-inflammatory, and immunomodulatory properties [281,284,285].

*Lycium barbarum* has been shown to afford neuroprotection to RGCs in animal models of chronic and acute OHT without affecting IOP values. Mi and colleagues (2012) showed that in mice subjected to acute OHT, oral pretreatment with LBP improved the survival ratio of RGCs, reduced the thinning of retinal inner nuclear layers, and preserved the integrity of the blood–retinal–barrier (BRB). The prevention of vascular damage and the neuroprotection observed were ascribed to the downregulation of receptor for advanced glycation end products (RAGE) and their ligands (AGE), endothelin-1 (ET-1), amyloid β-peptide (Aβ), and the related signaling pathways [286]. In addition, He and colleagues (2014) suggested that the activation of the erythroid 2-related factor (Nrf2)/heme oxygenase-1 (HO-1) antioxidant pathway contributes to the protection afforded by LBP following acute OHT [287].

Chan and coauthors (2007) first demonstrated that oral administration of the aqueous extract of dried fruit of *Lycium barbarum* protects RGCs in an in vivo model of chronic OHT induced by argon laser photocoagulation; the dose–response showed a U-shaped curve with the maximum protection obtained at 1 mg/kg/die given for 21 days [288]. The same research group suggested that the observed neuroprotection was associated with the modulation of microglia activation in the inner retina [289] and might involve the direct upregulation of neuronal crystallins, especially βB2-crystallin [290].

While the previous studies demonstrated the benefits of LBP applied before the OH induction (pretreatment), Lakshmanan and colleagues (2019) recently assessed the effects of the treatment when this was applied after the induction of chronic OHT (post-treatment). LBP post-treatment preserved RGC density and function. Pre and post-treatment intervention with LBP prevented RGC loss and preserved retinal structure and function, although pretreatment offered superior neuroprotection [291].

The neuroprotective effects of LBP have also been reported in IOP-independent glaucoma models. In rats that had undergone partial optic nerve transection (PONT) feeding with LBP, for 7 days before the insult, there was delayed secondary RGC degeneration and improved retinal function by inhibiting oxidative stress, increasing the JNK/c-jun pathway and modulating the activation of microglia/macrophages in the retina [292,293]. More recently, the neuroprotection afforded by LBP in the PONT model has been associated with the increase of macrophage M2 polarization and the reduction of the autophagy marker LC3II [294].

While LBP seems promising in preclinical animal studies, its effects on patients with ocular pathologies remain unknown. However, a recent double-masked clinical trial in retinitis pigmentosa patients showed that a daily supplementation with *Lycium barbarum* for 12 months preserved macular thickness and visual acuity and, therefore, supported the data reported in preclinical studies regarding the potential retinal neuroprotective effect of *Lycium barbarum* [295].

## 14. Saffron

Saffron is the dried red stigmas of *Crocus sativa L.* flower, which is a perennial stemless herb belonging to the Iridaceae family. Saffron is widely used in food preparation as a coloring and flavoring spice, and it is also long been used in traditional medical practice for its anticatarrhal, expectorant, eupeptic, anti-spasmodic, emmenagogue, and nerve-sedative properties [296].

Saffron stigmas contain more than 150 volatile and non-volatile active compounds [297]; safranal is the main component of the volatile fraction, while non-volatile constituents comprise crocins, crocetin, picrocrocin, and flavonoids (quercetin and kaempferol); see [298]. Crocin and crocetin (an aglycone of crocin) are potent antioxidants; however, their protective activities may rely on more complex mechanisms involving the modulation of gene expression. Nam et al. (2010) showed that crocin inhibits NF-kB activation and suppresses the production of nitric oxide (NO), TNF-α, IL-β, and ROS from activated microglial cells [299,300]. Several studies reported the beneficial effects of saffron extract or its components in in vivo and in vitro models of retinal degeneration. Protective effects of orally administered crocetin were reported in mice subjected to retinal ischemia, and these were mediated by the reduction of mitogen-activated protein kinases activation (p38, JNK) and redox-sensitive transcription factors (i.e., c-Jun and NF-kB) [301]. Oral administration of crocetin significantly protected against light-induced retinal damage [302], reduced retinal edema in a mouse model of retinal vein occlusion [303], and prevented inner retinal damage induced by an intravitreal injection of NMDA in mice via inhibition of the caspase pathway (preventing the activation of caspase-3 and caspase-9) [304].

Crocin analogs were found to raise blood flow in the retina and choroid of rabbits subjected to acute IOP, thus improving oxygenation and nutrient supply and facilitating retinal function recovery [305]. An intraperitoneal injection of crocin significantly enhanced RGC survival in a rat model of retinal ischemia/reperfusion injury through the activation of the PI3K/Akt signaling pathway [306]. In the same model of retinal damage, prevention of RGC death by crocin (given intraperitoneally) was associated with the upregulation of antioxidant enzymes (i.e., GSH and SOD), reduction of ROS and malondialdehyde (MDA) levels, and decreased phosphor-extracellular signal-related kinase (p-ERK) expression [307].

A recent study showed that oral administration of a hydrophilic saffron extract standardized to 3% crocin prevented RGC death and repressed retinal microglial activation in a mouse model of laser-induced OHT [308].

Despite the preclinical data, the clinical significance of saffron or its components in glaucoma patients is yet to be proven, and only a pilot clinical study has been published showing that oral saffron supplementation exerts a significant hypotensive effect in patients with primary open angle glaucoma [309].

## 15. Erigeron breviscapus

*Erigeron breviscapus* (vant.) Hand.Mazz. (EBHM) is a medicinal plant endemic in southwestern China. EBHM has been used to treat heart disease, cerebral infarction, digestive disorders, and apoplexy [310]. The major active components are scutellarin, a flavone glucuronide (5,6,4′-trihydroxyflavone-7-O-glucuronide), 3,5-dicaffeoylquinic acid, and erigoster B [311]. It has been shown that EBHM is endowed with neuroprotective effects. EBHM extract prevented neuronal apoptosis induced by transient focal ischemia [312], and scutellarin alleviated motor deficits caused by multiple sclerosis by inhibiting the apoptosis of neural stem cells and promoting their differentiation into myelin-producing oligodendrocytes [313]. Recently, the neuroprotective effects of EBHM have been investigated in experimental models of retinal degeneration. It has been shown that EBHM improved the ON axoplasmatic transport altered by acute IOP elevation [314] and prevented RGC death induced by ON crush in rats [315]. Visual function improvement by restoring multifocal electroretinogram (mfERG) altered by elevated IOP in rats was reported following treatment with EBHM [316].

Zhu and colleagues (2018) demonstrated that EBHM extract increased the survival of RGCs and inhibited the abnormal activation of microglia under acute elevated IOP conditions; the authors also reported in vitro and in vivo evidence supporting the hypothesis that scutellarin protects against hypoxic retinopathy via the inhibition of NLRP3 inflammasome signaling pathway [317].

Interestingly, the visual field protective effects of EBHM (given orally three times a day for a period of 6 months) were reported following a randomized, double blind, clinical trial in glaucoma patients with pharmacologically controlled IOP; however, further studies are needed to determine the safety and effectiveness of long-term treatment [318].

## 16. Conclusions

The rationale behind the use of nutritional supplementation in glaucoma is sustained by a significant amount of literature showing that natural compounds endowed with antioxidant, anti-inflammatory, and anti-apoptotic activities are effective in preventing RGC death in in vitro and in vivo models of retinal degeneration. However, the clinical significance of these data is yet to be proven. Indeed, in the available clinical studies, the small number of included patients, the study design heterogeneity, and the short follow-up period make it arduous to establish the clinical benefit of the treatment, and caution is needed in interpreting the results. Nevertheless, although further investigations are needed to determine their efficacy and safety, nutritional supplementation may represent an important coadjuvant in the therapeutic management of glaucoma.

## Figures and Tables

**Table 1:** Main sources of vitamins.

Vitamins		Sources
A (retinol)		Plant products: dark green leafy vegetables (i.e., spinach, broccoli), carrot, tomato, cabbage, winter squash, sweet potato, water melon, cantalope, deep orange fruits (i.e., papaya, mango, apricot);
		Animal products: butter, cream cheese, egg, cod liver oil, beef, liver, salmon
B Complex	B1 (thiamin)	Plant products: cereal grain, nut, bean, cauliflower, asparagus, orange, potato, brown rice, winter squash;
		Animal products: meat (i.e., pork, beef); liver, egg, fish (i.e., tuna, trout, seafood)
	B2 (riboflavin)	Plant products: cruciferous vegetables (i.e., broccoli, brussels sprout, spinach), mushroom, almond, nut, avocado;
		Animal products: milk, cheese, egg, red meat (i.e., beef), chicken
	B3 (niacin)	Plant products: date, nut, peanut, cereal grain, beans, tofu, pumpkin;
		Animal products: meat (i.e., pork, beef, lamb), liver, chicken, fish (i.e., tuna, salmon, sardine), egg
	B6 (pyridoxine)	Plant products: banana, chickpea, potato, sweet potato, tofu, pistachio, avocado;
		Animal products: meat (i.e., pork, beef), chicken, fish (i.e., tuna, snapper)
	B9 (folate)	Plant products: cereal grain, dark green leafy vegetables (i.e., spinach, romaine lettuce, asparagus, broccoli, brussels sprout), nut, peas, bean, avocado, mango, orange; Animal products: egg, liver, seafood
	B12 (cobalamin)	Animal products: fish (i.e., trout, salmon, tuna, clam), red meat (i.e., beef), liver, ham, egg, cheese
C (L-ascorbic acid)		Plant products: green leafy vegetables (i.e., broccoli, brussels sprout, spinach), cauliflower, green and red peppers, winter squash, tomato, sweet and white potatoes, many fruits (i.e., papaya, kiwi, orange, strawberries)
D (cholecalciferol)		Plant products: cereals, mushroom,
		Animal products: fish (i.e., salmon, sardine, herring, mackerel), cod liver oil, egg yolk, red meat, liver;
		Sunlight exposure
E (tocopherol)		Plant products: nuts (especially almond), sunflower oil and seeds, soybean oil, avocado, beet greens, collard greens, spinach, pumpkin, red bell pepper, asparagus, mango, avocado
